# Endofungal *Mycetohabitans rhizoxinica* Bacteremia Associated with *Rhizopus microsporus* Respiratory Tract Infection

**DOI:** 10.3201/eid2810.220507

**Published:** 2022-10

**Authors:** Shangxin Yang, Victoria Anikst, Paul C. Adamson

**Affiliations:** David Geffen School of Medicine at University of California–Los Angeles, Los Angeles, California, USA

**Keywords:** *Mycetohabitans rhizoxinica*, *Rhizopus microspores*, *Burkholderia rhizoxinica*, bacteremia, endofungal bacteria, mucormycosis, bacteria, fungi, respiratory infection, United States

## Abstract

We report *Mycetohabitans rhizoxinica* bacteremia in a 65-year-old woman in California, USA, who was undergoing chimeric antigen receptor T-cell therapy for multiple myeloma. Acute brain infarction and pneumonia developed; *Rhizopus microsporus* mold was isolated from tracheal suction. Whole-genome sequencing confirmed bacteria in blood as genetically identical to endofungal bacteria inside the mold.

*Mycetohabitans rhizoxinica* (previously *Burkholderia rhizoxinica*) and *M.* *endofungorum* are endofungal bacteria inhabiting *Rhizopus microsporus* mold. These bacteria form symbiosis to help the mold infect plants and produce mycotoxins, such as rhizoxin, that cause rice seedling blight (*1*,*2*). Genetically, *Mycetohabitans* species are highly similar to *Burkholderia* species but with significantly smaller genome sizes (3.3–3.8 Mb vs. 5.8–11 Mb), reflecting their endosymbiotic nature (*3*). *R. microsporus* causes mucormycosis, a devastating invasive fungal infection seen most prevalently in immunocompromised patients, but no strong evidence has suggested that the symbiont bacteria contribute to the pathogenicity of *R. microsporus* mold in humans (*1*,*4*). Isolation of endofungal bacteria have seldom been reported in clinical settings, most likely because of limitations in identification methods. In a study performed by the Centers for Disease Control and Prevention, *M. rhizoxinica* and *M. endofungorum* isolated from blood and wounds were characterized as oxidase-positive, gram-negative coccobacilli and could be reliably identified only by sequencing; no clinical information was provided to characterize the clinical presentation nor did researchers describe any link to *R. microsporus* (*4*). We report *M. rhizoxinica* bacteremia associated with multifocal pneumonia presumptively caused by *R. microsporus* mold in a severely immunocompromised patient.

## The Study

A 65-year-old woman in California, USA, with a history of relapsed and refractory multiple myeloma visited a hospital to receive chimeric antigen receptor (CAR) T-cell therapy. She previously had received 3 doses of the COVID-19 mRNA vaccine. On the day after CAR T-cell therapy, she experienced a rapid decline in mental status, accompanied by fever and hypotension, and was transferred to the intensive care unit. At that time, her total leukocyte count was 0.09 × 10^3^ cells/uL. Nasopharyngeal testing for COVID-19 by PCR was negative. She was diagnosed with immune effector cell-associated neurotoxicity syndrome and cytokine release syndrome. She received high doses of dexamethasone and 4 doses of tocilizumab, as well as anakinra, which was tapered over 7 days. The patient’s symptoms improved, and she was discharged after a 15-day hospital stay, with plans for a prolonged taper of orally administered dexamethasone (8 mg 2×/d). On discharge, the patient’s leukocyte count was 1.4 × 10^3^ cells/uL (87% neutrophils, 7% lymphocytes); the next day, her outpatient blood work showed mild lactic acidosis.

Four days after leaving the hospital, the patient sought treatment at an outpatient oncology clinic, reporting generalized weakness and fatigue. We performed blood and urine analyses and began a regimen of oral levofloxacin (500 mg daily). On day 6 after discharge, we noted a positive blood culture result, with a gram-negative rod. The urine culture grew 30,000 CFU/mL of *Klebsiella pneumoniae*. When evaluating the patient during clinical rounds, we performed repeat blood and urine cultures and recommended inpatient management, which was refused. We administered 1 intravenous dose of meropenem (1 g) at that visit and 1 dose of intravenous ceftriaxone (2 g) the next day. 

**Figure 1:**
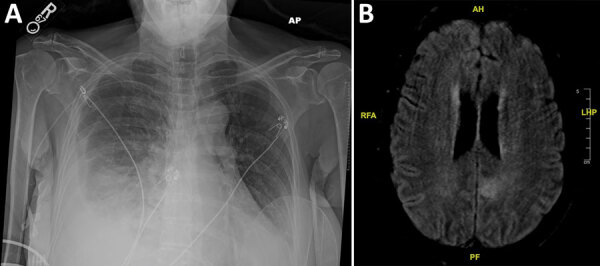
Imaging studies from a 65-year-old woman with multiple myeloma undergoing chimeric antigen receptor T-cell therapy who was admitted to the hospital for worsening foot and ankle pain, California, USA. A) Chest radiograph image, showing a moderate right-sided pleural effusion and adjacent pulmonary opacities indicative of pneumonia. B) Magnetic resonance imaging of the patient’s brain, showing the infarct involving the left medial parietal lobe.

On day 8 after discharge, the patient sought emergency treatment for worsening foot and ankle pain. Her leukocyte count was 0.44 × 10^3^ cells/uL. Bacterial identification of the positive blood culture was still pending. Results of repeat blood and urine cultures and COVID-19 PCR testing (nasopharyngeal) were negative; chest radiograph showed right-, middle-, and lower-lobe airspace opacification (Figure 1, panel A). We began piperacillin/tazobactam and performed an arthrocentesis, which showed crystals but revealed a negative Gram stain. Shortly thereafter, the patient reported acute numbness and weakness of her right leg. She subsequently developed dyspnea, respiratory distress, and altered mental status. We intubated her and conducted magnetic resonance imaging, which showed an acute infarct of the left medial parietal lobe, with hemorrhagic transformation (Figure 1, panel B). We transferred the patient to the intensive care unit, where she was febrile (38.2°C) and required 2 vasopressors. We administered vancomycin, caspofungin, and isavuconazole. The next day, hypotension worsened, requiring 3 vasopressors. Leukocyte was 0.16 × 10^3^ cells/uL. On hospital day 3, we obtained a tracheal aspirate for cultural analysis. Later that day, pulseless electrical activity occurred; the patient suffered cardiac arrest and died.

The day after her death, results of analysis of tracheal aspirate obtained on hospital day 3 revealed a mold. The bacteria from the initial positive blood culture were gram-negative, oxidase-positive coccobacilli not identifiable by the Vitek MS system (bioMérieux, https://www.biomerieux.com). The specimen was automatically reflexed to a laboratory-developed whole-genome sequencing (WGS) identification test (*5*). Drug-susceptibility testing showed presumptive (due to lack of breakpoint) susceptibility to most drugs tested, including amoxicillin/clavulanate, ceftriaxone, piperacillin/tazobactam, carbapenems, gentamicin, ciprofloxacin, and trimethoprim/sulfamethoxazole (Table).

**Table:** Antibiotic susceptibility results for *Mycetohabitans rhizoxinica* isolate from a 65-year-old woman with multiple myeloma undergoing chimeric antigen receptor T-cell therapy admitted to the hospital for worsening foot and ankle pain, California, USA*

Antibiotic	MIC, µg/mL*
Amoxicillin/clavulanate	≤2
Ceftriaxone	≤1
Ceftazidime	≤0.5
Ceftolozane/tazobactam	≤0.5
Cefepime	≤0.5
Ceftazidime/avibactam	≤2
Ertapenem	≤0.25
Imipenem	≤1
Meropenem	≤0.25
Piperacillin/tazobactam	≤8
Amikacin	8
Gentamicin	≤1
Tobramycin	≤1
Ciprofloxacin	0.5
Levofloxacin	≤0.5
Trimethoprim/sulfamethoxazole	≤1/20

*Susceptibility testing was performed on broth-microdilution panels prepared in-house in accordance with CLSI guidelines from the Clinical and Laboratory Standards Institute (https://www.clsi.org).

**Figure 2:**
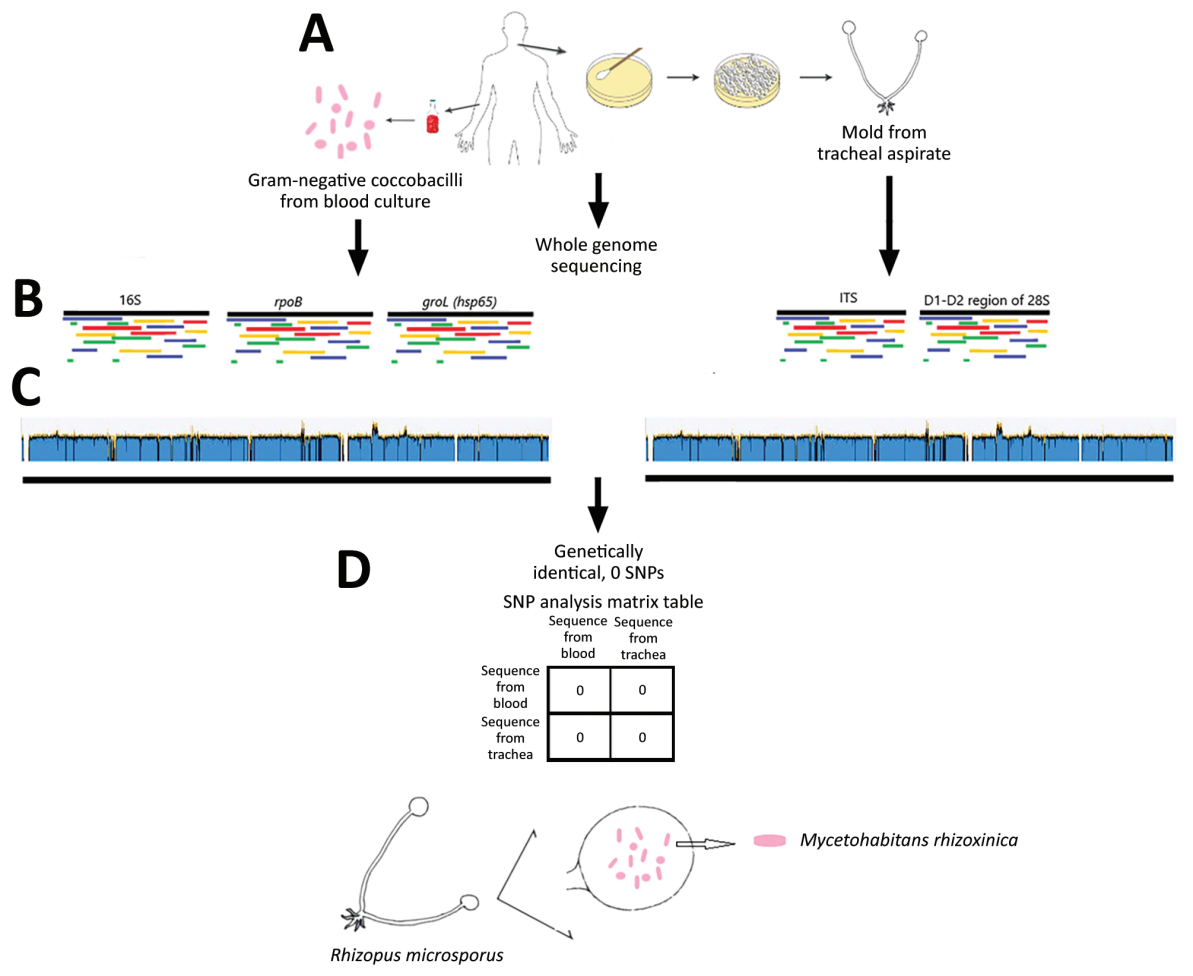
Whole-genome sequencing analysis of bacterial and fungal isolates (A) in a 65-year-old woman with multiple myeloma undergoing chimeric antigen receptor T-cell therapy, California, USA. The bacteria were identified as *Mycetohabitans rhizoxinica* with >99% identity in all 3 full-length marker genes compared with the reference organism (B, left side). The mold was identified as *Rhizopus microsporus* with >99% identity in the ITS gene and the D1–D2 region of the 28S gene compared with a reference organism (B, right side). Whole-genome sequences of the bacteria from blood (C, left side) revealed whole-genome coverage 94.0% and pairwise identity 95.5% with sufficient mean coverage of 298×. Whole-genome sequence of the mold from tracheal aspirate (C, right side) aligned to reference bacterial whole-genome sequence showed whole-genome coverage 94.1% and pairwise identity 95.8%, with sufficient mean coverage of 75×. The bacteria inside the mold from the trachea were genetically identical to the bacteria from the blood, as shown by the SNP analysis (D). ITS, internal transcribed spacer; SNP, single nucleotide polymorphism.

We performed WGS on the bacteria from both the blood and the mold obtained from tracheal suction using Illumina MiSeq (Illumina, https://www.illumina.com) (*5*) (Figure 2). We identified the bacteria as *M. rhizoxinica*, with >99% identity in all 3 full-length marker genes, including 16S, *rpo*B, and *gro*L (*hsp65*) compared with the type strain *M. rhizoxinica* HKI 454 (GenBank accession no. NC_014722.1). We identified the mold as *R. microsporus*, with >99% identity in the internal transcribed spacer (ITS) gene and the D1–D2 region of the 28S gene compared with *R. microsporus* var. chinensis CBS 631.82 (accession no. NR_149337.1 for ITS and HM849668.1 for D1–D2). We mapped the whole genome sequences of the bacteria and mold isolates (Genbank Sequence Read Archive data: PRJNA857096) to the *M. rhizoxinica* HKI 454 complete genome using Geneious Prime (Geneious, https://www.geneious.com) and achieved similar whole-genome coverage (bacteria, 94.0%; mold, 94.1%) and pairwise identity (bacteria, 95.5%; mold, 95.8%), with sufficient mean coverage (bacteria, 298×; mold, 75×). We used the mapped sequence reads from the bacteria and the mold for single-nucleotide polymorphism analyses using CLC Genomics Workbench (QIAGEN, https://www.qiagen.com) as previously described (*6*). Results showed no single-nucleotide polymorphism between the sequences in the bacteria from blood and the bacteria within the mold, indicating the bacteria in the blood was derived from the mold (Figure 2). Further genomic analysis confirmed the presence of a gene cluster (*rhi*A–*rhi*I) that encodes the biosynthesis of rhizoxin in both bacterial and mold isolates (*7*).

## Conclusions

Using WGS, we present clear evidence linking the endofungal bacteria *M. rhizoxinica*, isolated from a patient’s blood, to the bacteria residing within the *R. microsporus* mold isolated from the patient’s respiratory sample. The bacteria were isolated in the blood culture before initiating antibiotics were pansusceptible to the antibiotics tested, and cleared quickly after treatment. However, it is not clear whether the antibiotics retained activity against the bacteria within the fungal cytoplasm or whether activity was preserved for only bacteria outside the fungi. We hypothesize that the bacteria were most likely inside the mold in vivo and freed only after sample collection and then grew in the blood culture bottle during the incubation, when the fungus became degraded or lysed. Blood culture has low yield for Mucorales species (*8*), indicating those molds often die during the blood culture process. Because of the clinical manifestation, we believe the bacteria did not contribute to sepsis but rather served as a signal for a developing invasive mold infection that manifested during the immune suppression related to the patient’s CAR T-cell therapy. Unfortunately, the patient decompensated rapidly and died before the mold was identified and before we could initiate aggressive antifungal treatment.

Infarction and necrosis of infected tissues are hallmarks of mucormycosis (*9*). Disseminated mucormycosis, the presumptive diagnosis in this case based on the pulmonary findings and hemorrhagic brain infarction, is a rapidly progressive infection associated with a mortality rate >90% (*10*). This patient was at risk for disseminated mucormycosis because of profound immune suppression. Early diagnosis and prompt treatment of mucormycosis are key to improving clinical outcomes. Disseminated mucormycosis appears to be the underlying infectious process in this case, but this report lacks autopsy examination to confirm the presumptive diagnosis.

In conclusion, we found that isolation of endofungal bacteria *M. rhizoxinica* in the clinical setting might indicate invasive *Rhizopus* infection. Because identification methods used in most clinical laboratories are limited, endofungal bacteria may be underrecognized and require sequencing to identify. 
